# Attitudes toward COVID-19 vaccines in Chinese college students

**DOI:** 10.7150/ijbs.58835

**Published:** 2021-04-10

**Authors:** Wei Bai, Hong Cai, Shou Liu, Huanzhong Liu, Han Qi, Xu Chen, Rui Liu, Teris Cheung, Zhaohui Su, Chee H. Ng, Yu-Tao Xiang

**Affiliations:** 1 Unit of Psychiatry, Department of Public Health and Medicinal Administration, & Institute of Translational Medicine, Faculty of Health Sciences, University of Macau, Macao SAR, China; 2 Centre for Cognitive and Brain Sciences, University of Macau, Macao SAR, China; 3 Institute of Advanced Studies in Humanities and Social Sciences, University of Macau, Macao SAR, China; 4 Department of Public Health, Medical College, Qinghai University, Xining, Qinghai province, China; 5 Department of Psychiatry, Chaohu Hospital of Anhui Medical University, Hefei, Anhui Province, China; 6 The National Clinical Research Center for Mental Disorders & Beijing Key Laboratory of Mental Disorders Beijing Anding Hospital & the Advanced Innovation Center for Human Brain Protection, Capital Medical University, School of Mental Health, Beijing, China; 7 School of Nursing, Hong Kong Polytechnic University, Hong Kong SAR, China; 8 Center on Smart and Connected Health Technologies, Mays Cancer Center, School of Nursing, UT Health San Antonio, San Antonio, Texas, USA; 9 Department of Psychiatry, The Melbourne Clinic and St Vincent's Hospital, University of Melbourne, Richmond, Victoria, Australia

**Keywords:** Coronavirus disease 19, Vaccine, College students, Attitude

## Abstract

**Background:** Vaccination is an important preventative measure against the coronavirus disease 19 (COVID-19) pandemic. To implement vaccination and immunization programs effectively, it is essential to investigate public attitudes toward COVID-19 vaccines. This study examined the attitudes of Chinese college students toward COVID-19 vaccines and their associated factors. **Methods:** A cross-sectional study was conducted in college students nationwide from December 27, 2020 to January 18, 2021. Attitudes toward COVID-19 vaccines and acceptance of future vaccination programs were assessed. **Results:** Totally, 2,881 college students participated in this survey; of them, 76.3% (95% CI: 74.8% - 77.9%) were willing to accept a COVID-19 vaccine in the future. Multiple logistic analysis revealed that students living in urban (OR=1.409, 95% CI: 1.152 - 1.724, p=0.001) and those studying health-related courses (OR=1.581, 95% CI: 1.291 - 1.935, p<0.001) were more likely to have a positive attitude toward COVID-19 vaccines. In addition, those who were worried about being infected with COVID-19 (very much vs no, OR=1.690, 95% CI: 1.212-2.356, p=0.002), heard previously about COVID-19 vaccines (OR=1.659, 95% CI: 1.268-2.170, p<0.001), believed that vaccines are safe (Yes vs No, OR=3.570, 95% CI: 1.825-6.980), thought that vaccines can protect people from being infected with COVID-19 (Yes vs No, OR=1.957, 95% CI: 1.286-2.979, p=0.002), and had encouraged their family and friends to have a vaccine (Yes vs No, OR=17.745, 95% CI: 12.271-25.660, p<0.001) had higher acceptance of COVID-19 vaccination. **Conclusions:** A high rate of acceptance of COVID-19 vaccines was found among Chinese college students. However, vaccine uptake may be reduced by concerns about vaccine safety and efficacy. Alleviating these concerns and enhancing public confidence in vaccines are crucial for future immunization programs against the COVID-19 pandemic.

## Introduction

The coronavirus disease 2019 (COVID-19) emerged as a pandemic and a serious public health threat in 2020 1-3. As of late January, 2021, there had been more than 90 million cases and over 2 million deaths caused by COVID-19 4. Of the range of measures combating the pandemic 5, immunization is one of the most cost-effective preventive interventions 6. Many countries have accelerated vaccine research and developed vaccination programs against COVID-19; as of early 2021, there were more than 170 vaccines in pre-clinical development and over 60 vaccines in clinical development 7.

Although vaccine research has progressed very rapidly, public acceptance of and negative attitudes toward COVID-19 vaccines are significant challenges. Willingness to accept a vaccine against COVID-19 is recognized as a key issue in determining the success of a vaccination program 8. As it is important to examine public acceptance of vaccines, previous studies have examined the acceptance rate of the 2009 HIN1 influenza vaccine. For example, during the 2009 A/H1N1 pandemic, the vaccine acceptance rates ranged from 50% to 64% among adults in the USA 9-11. In China, over 60% of study respondents had intended to receive vaccination 12. Recent studies that examined the acceptance rate of COVID-19 vaccines found rates ranging from 23% to 91% among American medical students and adults, and Chinese adults 8, 13-16. Other associated factors of vaccine acceptability are also important to implement a vaccination strategy. For instance, a meta-analysis 17 of 126 studies on the moderating factors of the influenza vaccination program in China found that those having higher school education level, perceiving the vaccines were safe and effective, COVID-19 as a severe disease, receiving recommendations from healthcare workers, and receiving previous influenza vaccination were associated with better vaccination coverage. In addition, previous studies found that less severe depressive symptoms and more severe anxiety symptoms were associated with higher vaccine acceptance 18, 19.

The associated factors of COVID-19 vaccine acceptability in different populations have also been studied 8, 13, 16. For instance, attitudes toward vaccines (i.e. perceived safety and effectiveness of COVID-19 vaccines) were associated with vaccine acceptability in the general population of the USA 13, while similar results were also found in Japan and China 8, 16. Certain sub-populations of interest have an increased risk of contagion, such as college students who often live and study in crowded settings 20. Compared to other sub-populations, college students are better educated, more open-minded, and respond more quickly to public health issues, therefore, their attitudes toward COVID-19 vaccines are probably different 21. A study conducted among medical students in the USA found that concerns about vaccine safety/efficacy were associated with vaccine acceptability 15. In contrast, little is known about attitudes toward COVID-19 vaccine in students in other disciplines. In addition, no studies to date have examined the acceptance of COVID-19 vaccines among Chinese college students. Therefore, the aim of this study was to examine the attitude toward COVID-19 vaccines and its associated factors in Chinese college students.

## Methods

### Study settings and sample

This was a cross-sectional study conducted among college students nationwide from December 27, 2020 to January 18, 2021, using snowball sampling. Due to the risk of contagion of the COVID-19, face-to-face interviews were not adopted. Following previous studies 22-25, an online questionnaire was designed using the WeChat-based QuestionnaireStar application and subsequently a Quick Response code (QR code) was then generated. The QR code linked to the assessment tools was supported in several WeChat groups by influential academic staff including University Presidents, Faculty Deans and Department heads, who forwarded the QR code to students in their universities to encourage students to participate in this survey. WeChat is a widely used social communication application with more than 1 billion users in China. Participants who met the following criteria were included: (1) undergraduate students aged between 16 and 30 years; (2) Chinese ethnicity; (3) able to understand the purpose and contents of the assessment. To avoid missing any items in the questionnaire, all questions were set as mandatory to respond. This study was approved by the Institutional Review Board (IRB) of Beijing Anding Hospital. Online written informed consent was obtained. For those younger than 18 years, online written informed consent was provided by their guardians.

### Measures

Basic sociodemographic characteristics and health related information were collected, such as age, gender (female/male), academic grade (first/second/third/fourth/fifth year), academic course (health related/others), residence (urban/rural), and perceived health status (poor/fair/good).

Following a previous study on the influenza vaccine 26, several standardized questions were used to measure attitudes (5 items) and behavior (1 item) toward COVID-19 vaccines. The questions about attitudes toward COVID-19 vaccines were as follows: (1) Do you worry about being infected with COVID-19? (No/Fair/Very much); (2) Have you heard of COVID-19 vaccines previously? (No/Yes; including any information about COVID-19 vaccines, including both positive and negative news, vaccine development, safety and efficacy of vaccine from various channels (e.g., radio, television, telephone)); (3) Do you think COVID-19 vaccines could protect you from COVID-19? (No/Yes); (4) How safe do you think COVID-19 vaccines are? (They are not safe with obvious side effects /Not sure /They are safe with no side effects); (5) Will you encourage your family and friends to receive a COVID-19 vaccine? (No/Yes). The question about behavior towards COVID-19 vaccines: “Do you intend to have a COVID-19 vaccine in the future? (No/Yes)”.

The reliability and validity of the Patient Health Questionnaire - 9 (PHQ-9) (i.e. Cronbach's α coefficient =0.854) 27 and the Generalized Anxiety Disorder Assessment - 7 (GAD-7) (i.e. Cronbach's α coefficient=0.898) 28 questionnaire have been well validated in Chinese populations. The Chinese version of the PHQ-9 was used to assess severity of depressive symptoms 27, 29, which assesses the 9 Diagnostic and Statistical Manual of Mental Disorders-IV (DSM-IV) criteria for major depression as “0” (not at all) to “3” (nearly every day). The PHQ-9 total score ranges from 0 to 27, with a higher score indicating more severe depressive symptoms. Anxiety symptoms were measured using the Chinese version of the GAD-7 28, 30, which consists of 7 items with each scoring from 0 (not at all) to 3 (nearly every day). The total score ranges from 0 to 21, with a higher score indicating more severe anxiety symptoms.

### Statistical analysis

Data analyses were done with the SPSS version 24.0 (SPSS Inc., Chicago, Illinois, USA). Normal distributions were checked for continuous variables using P-P plots and one-sample Kolmogorov-Smirnov tests. Comparisons of socio-demographic and COVID-19 vaccine related variables between college students who would accept future COVID-19 vaccination and those who would not accept were conducted using chi-square tests, independent samples t-tests, and Mann-Whitney U tests, as appropriate. Binary logistic analysis with the “enter” method was used to examine the independent correlates of acceptance of future COVID-19 vaccination, with those that significantly differed in univariate analyses as independent variables. Odds ratio (OR) and corresponding 95% confidence intervals (CIs) were used to present results. Significantly statistical difference was set at 0.05 (two-tailed).

## Results

### Participant characteristics

A total of 2,892 college students were invited to participate in this survey, of whom, 2,881 college students met the study entry criteria and completed the assessment, giving a participation rate of 99.6%. The mean age was 19.83 years and 1,920 (69.0%, 95% CI: 67.4% - 70.8%) were females. The percentage of those who would accept future COVID-19 vaccination was 76.3% (95% CI: 74.8% - 77.9%) and a total of 592 (21.3%, 95% CI: 19.8% - 22.8%) students thought that COVID-19 vaccines were safe with no side effects.

**Figure 1:**
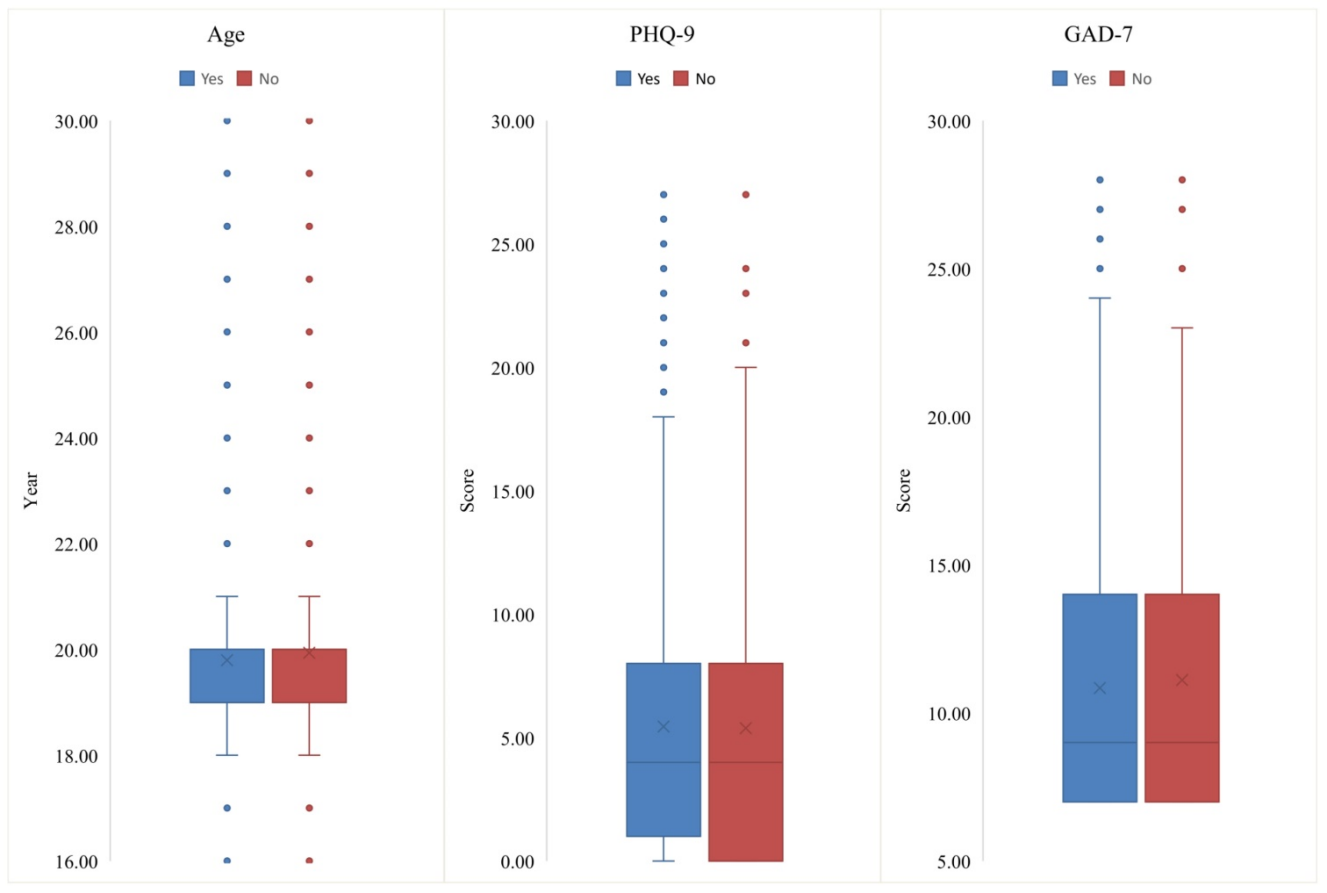
Box plots of age, the PHQ-9 and GAD-7 total scores between students who would and would not accept COVID-19 vaccination. COVID-19: Corona Virus Disease 2019; PHQ-9: 9-item Patient Health Questionnaire; GAD-7: 7-item Generalized Anxiety Disorder Scale

Table 1 shows the socio-demographic characteristics and health related information of the whole sample and comparisons between students who would and who would not accept future COVID-19 vaccines. No associations of depression (p=0.128) and anxiety (p=0.598) with COVID-19 vaccine acceptance were observed. There were significant differences in terms of residence (χ^2^=10.314, *p*=0.001) and health related courses (χ^2^=56.403, *p*<0.001) between the two groups. Figure 1 presents the distributions of continuous variables (e.g., age, and PHQ-9 and GAD-7 total scores) by attitudes toward COVID-19 vaccine. Responses to all the five questions about attitudes toward COVID-19 vaccines were significantly different between the two groups (all *p* values<0.05).

### Independent correlates of positive attitude toward future COVID-19 vaccine

**Figure 2:**
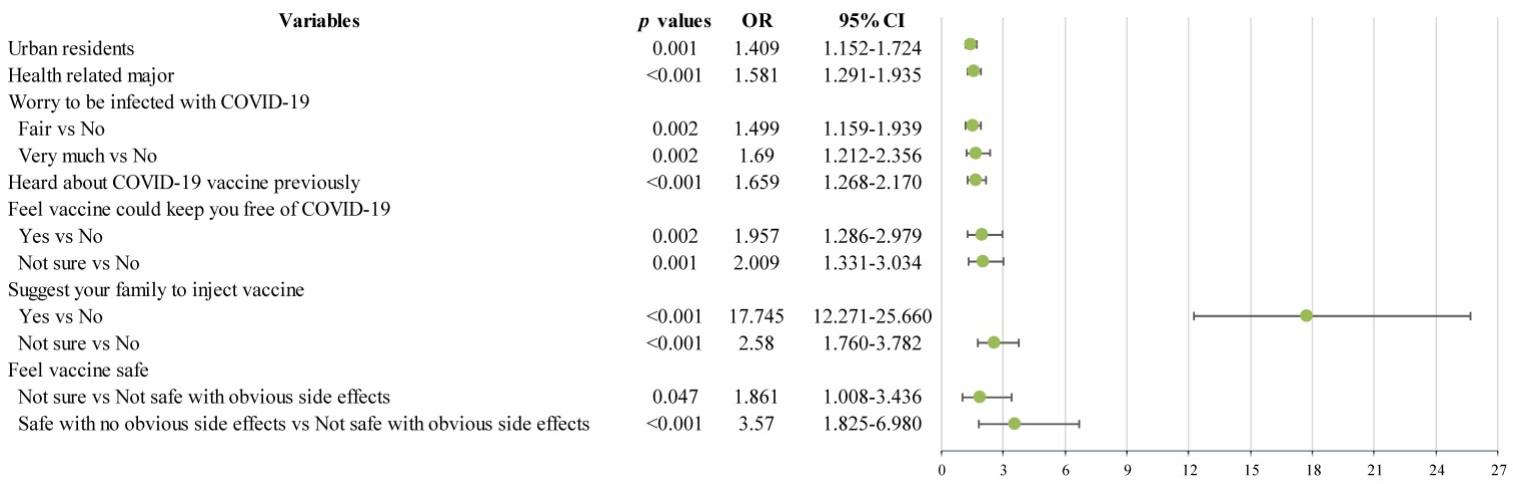
Independent correlates of willingness to accept future COVID-19 vaccine injection by binary logistic regression analysis. OR: odds ratio; CI: confidence interval; COVID-19: Corona Virus Disease 2019

Binary logistic regression analysis revealed that students living in urban and those with health-related course were positively associated with acceptance of future COVID-19 vaccination (Figure 2). In addition, college students who worried about being infected with COVID-19, who heard about the COVID-19 vaccines previously, who thought that vaccines are safe, who thought that vaccines could protect people from being infected with COVID-19, and who encouraged their family and friends to have the vaccine (all *p* values<0.05) were more likely to accept future COVID-19 vaccination (Figure 2).

## Discussion

To the best of our knowledge, this was the first-ever study globally that examined the attitudes of university students toward COVID-19 vaccines and their associated factors. The response rate in this online study was very high (99.6%), probably because the QR code linked to the invitation and assessment tools was sent by influential university academic staff. In this study most of Chinese college students (76.3%; 95% CI: 74.8% - 77.9%) were willing to accept a COVID-19 vaccine in the future, which is similar to the corresponding figures during the 2009 A/H1N1 pandemic among college students in Macau, China (72.7%) 20, but higher than that in adults in the USA (from 50%-64%) 9-11 and in college students in Turkey (11.9%) 31. Compared to the results in Europe, our finding was lower than the COVID-19 vaccination acceptance rate among college students in Italy (86.1%) 21 but higher than that in Malta (44.2%) 32. Apart from different socio-demographic factors 33, possible reasons for the discrepancy between these studies include the different levels of health literacy particularly with regard to immunization programs, as well as local health policy and clinical practices 34. For instance, since early 2020, the Chinese government has adopted a range of strict containment measures (e.g., early identification and isolation of suspected and infected cases, travel restrictions, and school closure) 35, 36. This may increase the level of public awareness regarding the risk of COVID-19 in China, which may contribute to the relatively high acceptance of vaccination.

Certain socio-demographic characteristics were associated with acceptance of future COVID-19 vaccines among students. Compared with those from rural areas, college students from urban areas were more likely to accept a COVID-19 vaccine (OR=1.409, 95% CI: 1.152-1.754). In contrast, rural residents (82.2%) in China had a higher acceptance rate of vaccination against A/H1N1 than urban residents (55.3%) during the 2009 A/H1N1 pandemic 12, which is probably due to different access to relevant health information and healthcare service coverage 37. For example, both primary care and hospital-based services are insufficient in rural areas, hence rural residents may be more likely to accept preventive measures, such as vaccination, to reduce the risk of contracting infectious diseases. However, in China college students are covered by basic health insurance by universities and provided adequate health information. Therefore, the different acceptance rates of COVID-19 vaccines between rural and urban residents found in previous studies 12 could not be extrapolated to college students. We speculate that different attitudes toward COVID-19 vaccines between rural and urban students' parents and other family members 12 may in turn influence students' acceptance rate of COVID-19 vaccines.

In this study 79.1% of students studying health related courses were willing to get a COVID-19 vaccine, which is similar to the corresponding figure (77%) of a study conducted at medical schools in the US 15. The higher vaccine acceptance rate among Chinese college students in health-related courses (OR=1.581, 95% CI: 1.291-1.935) compared to those in other courses, could be attributed to higher awareness of the importance of COVID-19 vaccines in disease prevention 38. Moreover, many students in health-related courses often need to visit medical facilities, such as hospitals and primary care services. Therefore, they perceive their likelihood of infection is higher than other students, and a higher need for vaccination for their health protection. However, another study in Italy did not find any differences between healthcare and non-healthcare students 21. Such discrepancy may be due to different study periods reflecting different pandemic severity: Barello et al.'s study was conducted at the beginning of the COVID-19 pandemic, and ours was conducted in the later part of the pandemic.

In addition, we found that students who worried about being infected with COVID-19, who previously heard about COVID-19 vaccines, who thought that COVID-19 vaccines could provide protection, who thought that vaccines are safe, and who encouraged their family to get vaccine were more likely to get a COVID-19 vaccine in future. This is similar to the findings of previous studies on seasonal influenza vaccine in China 26, in which young workers' attitude and behavior toward seasonal influenza vaccine in south China were examined and variables “heard of the influenza vaccine” (OR=2.20, 95% CI: 1.08-4.48) and “believe that the influenza virus vaccine can protect from influenza” (OR=3.33, 95% CI: 1.16-9.55) were associated with high acceptance of the influenza vaccine 26. It should be noted that most of these factors were related to beliefs of vaccine safety and efficacy. Another study 39 conducted in healthcare workers in Belgium and Canada found that lack of acceptance was mostly driven by vaccine safety concerns. A study in the UK 40 also found that common concerns about COVID-19 vaccine safety and efficacy were the main determinants of vaccine acceptance. All findings suggest that public education on the efficacy and safety of the COVID-19 vaccine is important for future widespread use of the vaccine 14.

The strengths of this study are the large sample size and the coverage of all provinces in China. However, several limitations should be noted. First, this study is cross-sectional; hence the casual relationship between attitudes toward and acceptance of COVID-19 vaccines could not be examined. Second, some factors associated with vaccine acceptance, such as family income and past history of having influenza vaccine, were not recorded. Third, due to logistical reasons, random sampling cannot be adopted. Instead, following other studies 22, 23, snowball sampling method was used in this study, which may result in selection bias and poor representativeness. Finally, to date no standardized questionnaires on attitude toward COVID-19 vaccines have been developed. Instead, a selection of standardized questions was used to measure attitudes and behavior toward COVID-19 vaccines in this study.

In conclusion, high acceptance of COVID-19 vaccines was found among Chinese college students. However, vaccine uptake may be reduced by concerns about vaccine safety and efficacy. Alleviating these concerns and enhancing public confidence in COVID-19 vaccines are crucial for future vaccination strategies and immunization programs against the COVID-19 pandemic.

## Figures and Tables

**Table 1:** Demographic characteristics of participants

Variables	Total (N=2,881)		Acceptance of COVID-19 vaccination				χ^2^	df	*P*
			Yes (N=2,123)		No (N=758)				
	N	%	N	%	N	%			
Female gender	1,920	66.6	1418	66.8	502	66.2	0.080	1	0.777
Urban residents	1,322	45.9	1012	47.7	310	40.9	10.314	1	**0.001**
Health related course	1,711	59.4	1,378	63.5	363	47.9	56.403	1	**<0.001**
School grade							3.519	4	0.475
First year	1,651	57.3	1,229	57.9	422	55.7			
Second year	601	20.9	431	20.3	170	22.4			
Third year	305	10.6	224	10.6	81	10.7			
Fourth year	221	7.6	168	7.9	53	7.0			
Fifth year	103	3.6	71	3.3	32	4.2			
Perceived health status as good	2,174	75.5	1,594	75.1	580	76.5	0.621	1	0.431
Worried about being infected with COVID-19							13.025	2	**0.001**
No	518	18.0	349	16.4	169	22.3			
Fair	1,851	64.2	1,388	65.4	463	61.1			
Very much	512	17.8	386	18.2	126	16.6			
Heard about COVID-19 vaccines previously	2,464	85.5	1909	89.9	555	73.2	125.854	1	**<0.001**
Thought COVID-19 vaccines could provide protection							185.177	2	**<0.001**
No	199	6.9	86	4.1	113	14.9			
Yes	1,371	47.6	1,145	53.9	226	29.8			
Not sure	1,311	45.5	892	42.0	419	55.3			
Encouraged family to get vaccine							756.589	2	**<0.001**
No	223	7.8	48	2.3	175	23.1			
Yes	1,989	69.0	1,754	82.6	235	31.0			
Not sure	669	23.2	321	15.1	348	45.9			
Thought vaccines are safe							124.592	2	**<0.001**
									
Not safe with obvious side effects	70	2.4	27	1.3	43	5.7			
Not sure	2,219	77.0	1,570	74.0	649	85.6			
Safe with no obvious side effects	592	20.6	528	24.8	66	8.7			
									
	**Mean**	**SD**	**Mean**	**SD**	**Mean**	**SD**	**t/Z**	**df**	***P***
Age (Years)	19.83	2.02	19.80	2.06	19.93	1.90	1.664	1435.988	0.096
PHQ-9 total	5.43	5.45	5.45	5.35	5.38	5.74	-1.522	---^*^	0.128
GAD-7 total	10.88	4.72	10.84	4.61	10.99	5.06	-0.527	---^*^	0.598

Bolded values: <0.05; M: mean; SD: standard deviation; COVID-19: Corona Virus Disease 2019; PHQ-9: 9-item Patient Health Questionnaire; GAD-7: 7-item Generalized Anxiety Disorder Scale;^ *^ Mann-Whitney U test.
